# Clinical presentation and extent of resection impacts progression-free survival in spinal ependymomas

**DOI:** 10.1007/s11060-024-04623-4

**Published:** 2024-03-04

**Authors:** Mark A. Davison, Daniel T. Lilly, Arpan A. Patel, Ahmed Kashkoush, Xiaoying Chen, Wei Wei, Edward C. Benzel, Richard A. Prayson, Samuel Chao, Lilyana Angelov

**Affiliations:** 1 Department of Neurosurgery, Neurological Institute, Cleveland Clinic, Cleveland, OH USA; 2 Department of Quantitative Health Sciences, Lerner Research Institute, Cleveland Clinic, Cleveland, OH USA; 3 Department of Anatomic Pathology, The Robert J. Tomsich Pathology and Laboratory Medicine Institute, Cleveland Clinic, Cleveland, OH USA; 4 Rose Ella Burkhardt Brain Tumor & Neuro-Oncology Center, Cleveland Clinic, Cleveland, OH USA; 5 Cleveland Clinic Lerner College of Medicine of Case Western Reserve University, Cleveland, OH USA; 6 Neurologic Oncology and Radiosurgery Fellowships, Neurological Surgery, CCLCM at CWRU, Rose Ella Burkhardt Brain Tumor & Neuro-Oncology Center, 9500 Euclid Ave., CA-51, 44195 Cleveland, OH USA

**Keywords:** Spinal ependymoma, Survival, Progression-free survival, Surgical resection, Gross total resection

## Abstract

**Purpose:**

Primary treatment of spinal ependymomas involves surgical resection, however recurrence ranges between 50 and 70%. While the association of survival outcomes with lesion extent of resection (EOR) has been studied, existing analyses are limited by small samples and archaic data resulting in an inhomogeneous population. We investigated the relationship between EOR and survival outcomes, chiefly overall survival (OS) and progression-free survival (PFS), in a large contemporary cohort of spinal ependymoma patients.

**Methods:**

Adult patients diagnosed with a spinal ependymoma from 2006 to 2021 were identified from an institutional registry. Patients undergoing primary surgical resection at our institution, ≥ 1 routine follow-up MRI, and pathologic diagnosis of ependymoma were included. Records were reviewed for demographic information, EOR, lesion characteristics, and pre-/post-operative neurologic symptoms. EOR was divided into 2 classifications: gross total resection (GTR) and subtotal resection (STR). Log-rank test was used to compare OS and PFS between patient groups.

**Results:**

Sixty-nine patients satisfied inclusion criteria, with 79.7% benefitting from GTR. The population was 56.2% male with average age of 45.7 years, and median follow-up duration of 58 months. Cox multivariate model demonstrated significant improvement in PFS when a GTR was attained (*p* <.001). Independently ambulatory patients prior to surgery had superior PFS (*p* <.001) and OS (*p* =.05). In univariate analyses, patients with a syrinx had improved PFS (*p* =.03) and were more likely to benefit from GTR (*p* =.01). Alternatively, OS was not affected by EOR (*p* =.78).

**Conclusions:**

In this large, contemporary series of adult spinal ependymoma patients, we demonstrated improvements in PFS when GTR was achieved.

## Introduction

Spinal ependymomas are the most common glial spinal tumor in adults comprising up to 60% of intramedullary neoplasms [1, 2]. Ependymomas derive from the ependymal cell lining within the central spinal canal and filum terminale and are generally slow growing tumors [3] Primary treatment of intramedullary ependymomas involves surgical resection, particularly in patients presenting with mild or moderate neurologic deficits [4, 5]. Despite their relatively favorable prognosis in comparison to other intramedullary glial tumors, a considerable proportion of ependymomas recur, resulting in lifelong morbidity and mortality [6].

An extensive body of literature exists investigating clinical and treatment characteristics that portend improvements in overall survival (OS) and progression-free survival (PFS) metrics in spinal ependymoma patients [2, 4, 5, 7–15]. Furthermore, a number of authors have demonstrated the benefits of gross total resection (GTR) over subtotal resection (STR) as a principal treatment for spinal ependymomas using both OS and PFS as primary outcome criteria [4, 7, 9, 15]. Abdel-Wahab et al. conducted one of the largest retrospective reviews of 120 spinal ependymoma patients undergoing primary surgical treatment with or without adjuvant radiation from 1953 to 2000. The authors demonstrated that GTR was significantly related to PFS and OS through a univariate and multivariate model, respectively [8].

While the existing evidence is compelling, these studies are limited by either a relatively small sample size, insufficient follow-up durations, or outdated data which pre-date revolutionary innovations in neurosurgical care including the introduction of the operating microscope in 1957, the routine use of magnetic resonance imaging (MRI) for intramedullary neoplasms in 1983, and the application of motor evoked potentials to spinal surgery in 1989 [16]. Moreover, the recent upgrade of myxopapillary ependymomas to grade 2 lesions in the 2021 World Health Organization (WHO) classification update diminishes the utility of preceding investigations [17]. Survival studies inclusive of modern innovations and treatment paradigms are essential, as this data establishes a baseline for which to compare novel interventions and practices. Therefore, the objective of this study was to investigate the relationship between clinical presentation and extent of surgical resection with OS and PFS in a large, contemporary cohort of spinal ependymoma patients.

## Methods

This study was approved by our institutional review board.

### Study population

Adult patients (≥ 18 years) with a pathology-confirmed diagnosis of spinal ependymoma who underwent primary surgical management at our hospital from 2006 to 2021 were identified from the institutional spinal tumor board registry. Included patients were required to have pre- and postoperative MRI studies and at least 3 months of postoperative clinical follow-up with a documented neurologic examination. Patients were excluded if they underwent prior treatment for their spinal lesion at another institution or if their primary surgical management comprised an ependymoma lesion biopsy only. Moreover, patients with a diagnosis of subependymoma (WHO grade 1) were omitted as these pathologic subtypes exhibit distinctly different behavior and are unlikely to recur [18].

### Data collection

Patient charts were retrospectively reviewed for demographic data and preoperative symptoms, including the presence of motor, sensory, and urinary symptoms as well as ambulatory status. Ependymoma characteristics such as pathology, WHO grade, Ki-67 index, lesion location, number of vertebral body segments involved (i.e. lesion size), and presence of a syrinx were also collected. Lesion location was classified as cervical, cervicothoracic, thoracic, thoracolumbar, or lumbosacral & filum terminale. Extent of surgical resection was determined from postoperative contrasted MRI sequences, and was divided into 2 classifications: gross total resection (GTR), subtotal resection (STR). Patients were designated a subtotal resection if there was an attempted resection and evidence of residual tumor on the postoperative MRI. The Modified McCormik scale was used to track functional outcomes and was calculated preoperatively, at discharge from the index hospitalization, and at the 1-year postoperative time points. Additionally, clinical symptoms 1-year after surgery and the use of adjuvant radiation or chemotherapy was noted.

Tumor progression was defined as MRI evidence of progression or recurrence of a previously treated ependymoma. In patients without lesion recurrence, the date of last MRI was used to compute PFS. Time of latest clinical encounter was referenced to calculate OS in living patients, while documented date of death was used for patients with mortality.

### Statistical analysis

Patient characteristics were summarized using means and frequencies with percentages where appropriate. Fisher’s exact test and Wilcoxon rank sum test were used to compare patient characteristics between GTR and STR groups where applicable. Log-rank test was used to compare OS and PFS between patient groups. Cox proportional hazard model was used to associate multiple patient characteristics with PFS. Subgroup analyses of patients with the myxopapillary pathology subtype were performed. All tests were two-sided and p-values ≤ 0.05 were considered significant. Statistical analysis was performed using SAS Studio 3.7 (SAS Institute, Cary, NC) and R version 4.2 (R Foundation, Vienna, Austria).

## Results

A total of 149 patients were initially identified from our institutional tumor board registry, of which 80 were excluded for the following indications: previous surgical management at another institution (*n* = 37), non-operative management (*n* = 24), non-ependymoma or subependymoma pathologic diagnosis (*n* = 14), insufficient clinical follow-up (*n* = 3), surgical management comprised of biopsy only (*n* = 2). Therefore, 69 patients diagnosed with spinal ependymoma satisfied the inclusion criteria and were considered for analysis. Males comprised 56.5% of the cohort and the average patient age was 45.7 years. Radiographic GTR was achieved in 55 patients (79.7%), while 14 patients (20.3%) underwent an incomplete resection and comprised the STR cohort. Sensory deficit was the most commonly observed preoperative symptom (73.9%) and median preoperative McCormik scale was 2 (range 1–5). Most common ependymoma locations included cervical (27.5%), thoracic (24.6%), and cervicothoracic (18.8%). Comparing lesion characteristics by extent of resection, while the lesion sizes were similar amongst cohorts, the GTR group were more likely to be associated with a syrinx (GTR: 52.7%, STR: 14.3%; *p* =.01). Lesions situated more rostrally along the neuro axis were more likely to be resected completely. More specifically, 89.5% lesions involving exclusively the cervical spine and 100% of lesions involving the cervical and thoracic spine resulted in a GTR outcome. Ki-67 index was inconsistently documented and only available in 47 patients, where the average and median were 4% and 3% for the GTR cohort (*n* = 40), respectively while it was 14% and 3% for the STR patients (*n* = 7), respectively. Adjuvant radiation therapy was administered in 13 patients, of which one patient also received postoperative chemotherapy. Generally, adjuvant therapies were administered to patients with grade 3 pathology, higher ki-67 scores, and cases of incomplete surgical resection. In fact, 64.3% of patients with residual tumor following surgery compared to 7.3% of the GTR cohort underwent adjuvant radiation (*p* <.001), (Table 1).

**Table 1:** Patient demographics, preoperative symptoms, lesion characteristic, and treatment modifiers for total population, gross total resection (GTR) and subtotal resection (STR) cohorts, n (%)

	Total Population	GTR	STR	P-value
Patients	69	55	14	--
Males	39 (56.5)	32 (58.2)	7 (50.0)	0.76
Age (mean, years)	45.7	45.5	46.4	0.69
**Preoperative Symptoms**				
Urinary Symptoms	16 (23.1)	11 (20.0)	5 (35.7)	0.29
Sensory Symptoms	51 (73.9)	43 (78.2)	8 (57.1)	0.17
Motor Weakness	32 (46.4)	27 (49.1)	5 (35.7)	0.55
Independently Ambulatory	62 (89.9)	50 (90.9)	12 (85.7)	0.62
Preoperative McCormik Scale (mean)	1.8	1.9	1.6	0.28
**Lesion Characteristics**				
Number of vertebral body levels involved (mean)	2.7	2.5	3.2	0.28
Presence of Syrinx	31 (44.9)	29 (52.7)	2 (14.3)	**0.01**
**Lesion Grade**				
Grade 2	66 (95.7)	54 (98.2)	12 (85.7)	0.10
Grade 3	3 (4.3)	1 (1.8)	2 (14.3)	
**Lesion Location**				
Cervical	19 (27.5)	17 (30.9)	2 (14.3)	**--**
Cervicothoracic	13 (18.8)	13 (23.6)	0 (0.0)	
Thoracic	17 (24.6)	13 (23.6)	4 (28.6)	
Thoracolumbar	6 (8.7)	1 (1.8)	5 (35.7)	
Lumbar	8 (11.6)	6 (10.9)	2 (14.3)	
Lumbosarcal & Filum	6 (8.7)	5 (9.1)	1 (7.1)	
**Treatment Modifiers**				
Adjuvant Chemotherapy	1 (1.4)	0 (0.0)	1 (7.1)	0.20
Adjuvant Radiation Therapy	13 (18.8)	4 (7.3)	9 (64.3)	**< 0.001**

Median population follow-up duration was 58 months (range 8–209 months). Five patient deaths and 15 radiographic progression events occurred during the follow-up window. Population OS and PFS rates at 5-years were 95% (95% CI, 89-100%) and 70% (95% CI, 58-86%), respectively. Following surgery, 23 patients (33.3%) experienced an increase in their McCormik scale, however there was no difference in rate of increase between surgical cohorts (Table 2).

**Table 2:** Outcome metrics and postoperative symptoms for total population, gross total resection (GTR) and subtotal resection (STR) cohorts, n (%). P-values by Fisher’s exact test and log rank test

	Total Population	GTR	STR	P-value
Patients	69	55	14	--
**Outcome Metrics**				
Follow-up Duration (median, months)	58	58	52	--
Tumor Progression (events)	15	5	10	**< 0.001**
Patient Deaths (events)	5	3	2	0.27
5-year Progression-Free Survival (months, 95% CI)	70% (58–86%)	85% (73–100%)	18% (5–62%)	**< 0.001**
5-year Overall Survival (months, 95% CI)	95% (89–100%)	94% (86–100%)	100% (100–100%)	0.77
**Postoperative symptoms**				
Urinary Symptoms	12 (17.4)	9 (16.4)	3 (21.4)	0.70
Sensory Symptoms	53 (76.8)	43 (78.2)	10 (71.4)	0.72
Motor Weakness	36 (52.2)	29 (52.7)	7 (50.0)	1
Independently Ambulatory	47 (68.1)	38 (69.1)	9 (64.3)	0.76
Increased McCormik Scale	23 (33.3)	18 (32.7)	5 (35.7)	1

### Extent of resection and overall-survival

**Fig. 1:**
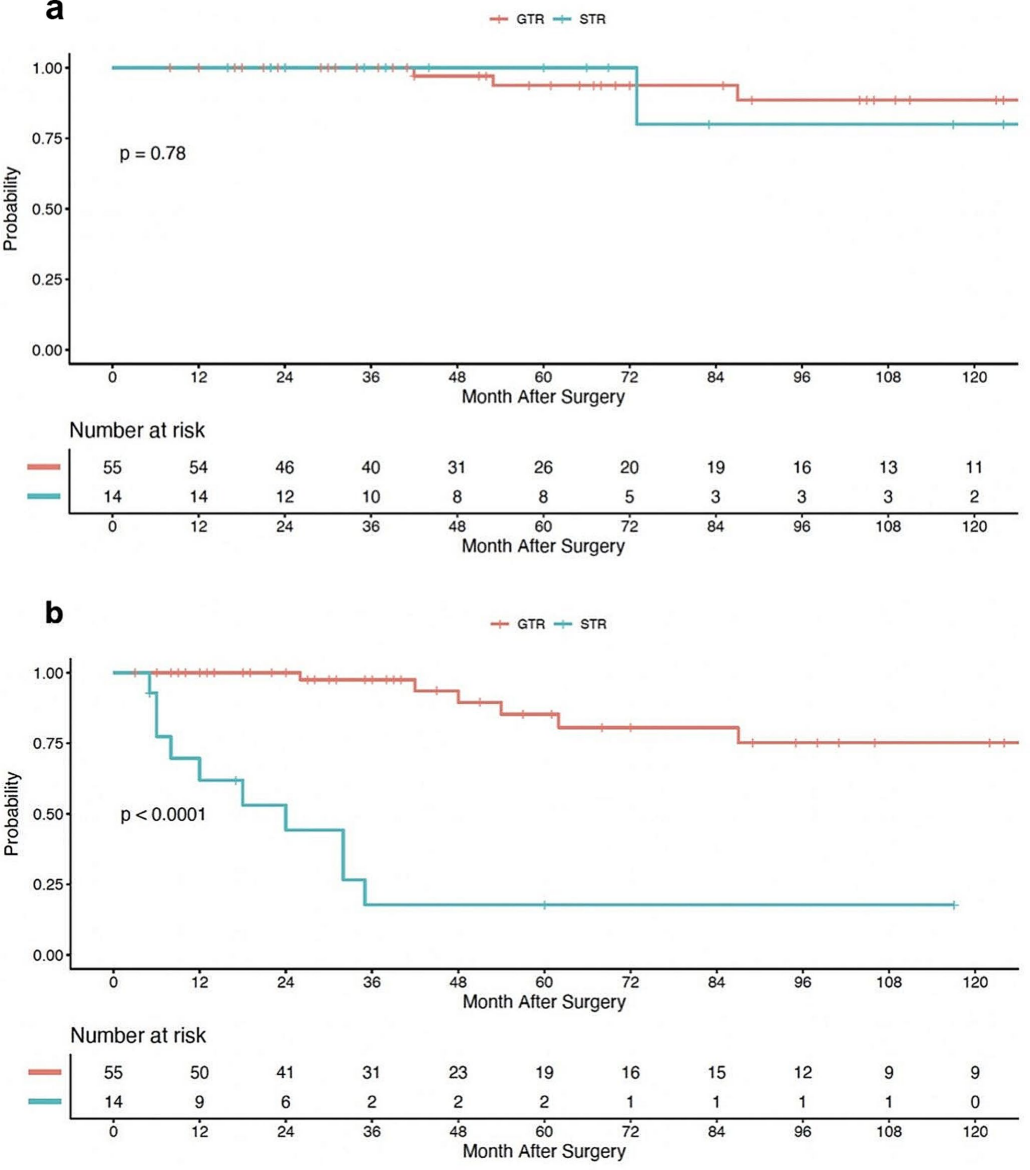
Kaplan-Meier curve for overall patient survival (**a**) and progression-free survival (**b**), stratified by extent of surgical resection

Overall survival data was limited by only 5 deaths observed in our series of 69 patients. In patients treated with GTR, OS rates at 2-, 5-, and 8-years were 100% (95% CI, 100%-100%), 94% (95% CI, 86-100%), and 89% (95% CI, 77-100%), respectively. Alternatively, in patients undergoing a STR, 2-, 5-, and 8-years OS rates were 100% (95% CI, 100%-100%), 100% (95% CI, 100%-100%), and 80% (95% CI, 52-100%), respectively. Log-rank test demonstrated no statistical difference in OS when stratified by extent of resection (*p* =.78), Fig. 1a. Moreover, patients without clinically detectable preoperative motor deficits and similarly, patients who were independently ambulatory prior to surgery were more likely to have superior OS (*p* =.04 and *p* =.05, respectively). Motor deficits 1-year post op did not have any effect on OS however (*p* =.22).

### Extent of resection and progression-free survival

Patients undergoing GTR had estimated 2-, 5-, and 8-year PFS rates of 100% (95% CI, 100%-100%), 85% (95% CI, 73-100%), and 75% (95% CI, 59-95%), respectively. Conversely in the STR cohort, the 2-, 5-, and 8-year PFS rates were 44% (95% CI, 23-83%), 18% (95% CI, 5–62%), and 18% (95% CI, 5-62%), respectively. Log-rank analysis demonstrated a significant improvement in PFS when a GTR was attained (*p* <.0001), Fig. 1b. Similarly, patients with a syrinx component to their pathology had improved PFS compared to patients without (*p* =.03). Furthermore, patients who were independently ambulatory (*p* <.001) and those without symptoms of urinary dysfunction (*p* <.001) prior to surgical resection had statistically superior PFS.

### Myxopapillary and non-myxopapillary pathology subgroup analysis

**Fig. 2:**
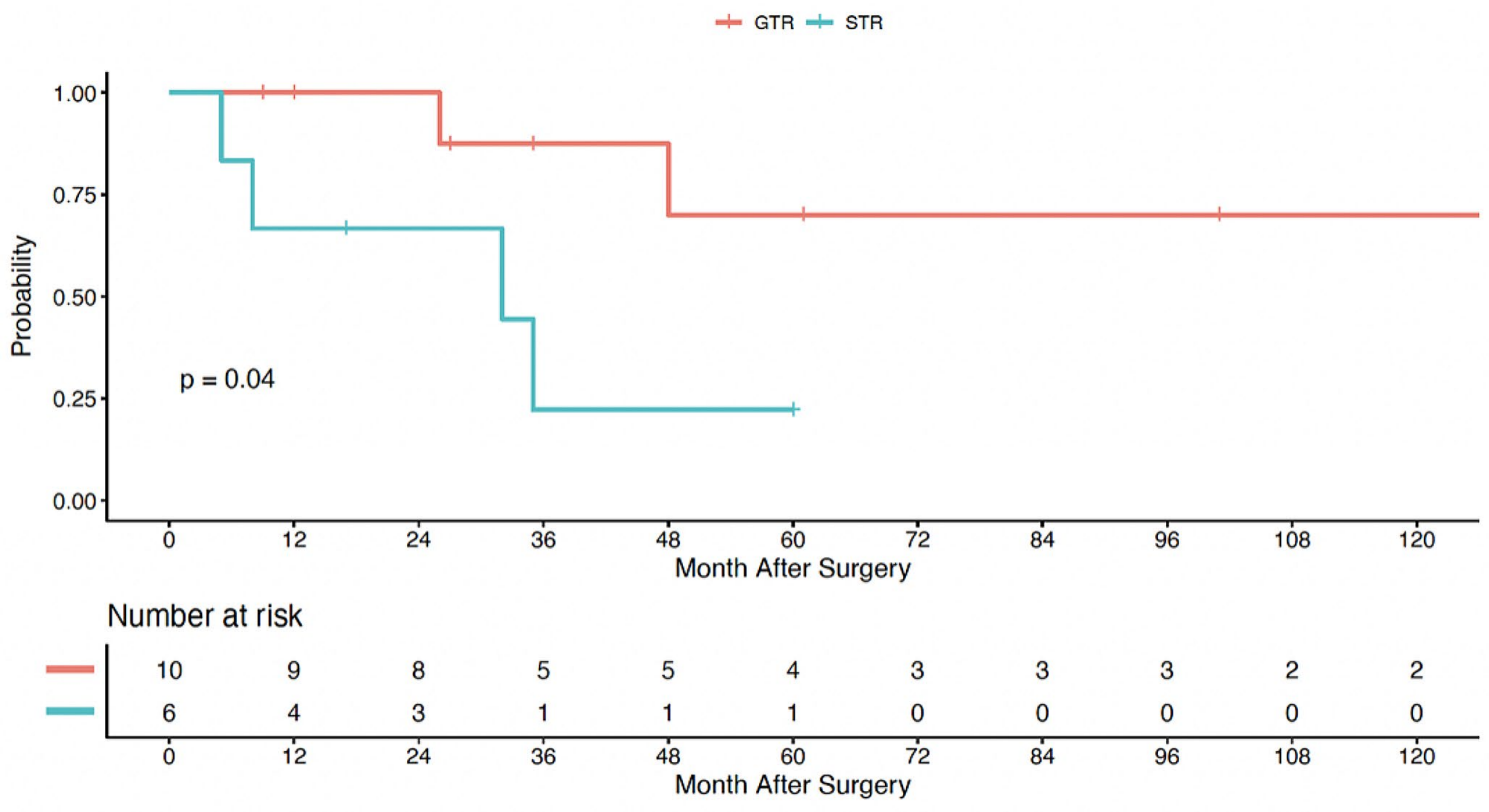
Kaplan-Meier curve for progression-free survival of patients with myxopapillary pathology, stratified by extent of surgical resection

Patients with a myxopapillary pathology comprised 23.2% (*n* = 16) of the study population. Of this subset of 16 patients, 37.5% experienced radiographic progression and 2 patients (12.5%) died during the study period. The OS and PFS were similar between patients with and without a myxopapillary diagnosis. While not a statistically significant finding, myxopapillary patients were less likely to benefit from a complete resection, with 84.9% of non-myxopapillary patients achieving GTR versus 62.5% in the myxopapillary cohort (*p* =.08). Adjuvant radiotherapy was more common in patients with a myxopapillary diagnosis (myxopapillary: 43.8%, non-myxopapillary: 11.3%; *p* =.008). The PFS rates of myxopapillary ependymoma patients undergoing GTR at 2- and 5-years were 100% (95% CI, 100%-100%) and 70% (95% CI, 42-100%), respectively. Alternatively, when only achieving STR, the PFS rates at 2- and 5-years dropped to 67% (95% CI, 38-100%), 22% (95% CI, 4-100%), respectively. Log-rank analysis demonstrated a significant positive effect of GTR on the PFS in the myxopapillary subgroup (*p* =.04), Fig. 2. Due to the infrequency of mortality events in this subgroup (*n* = 2), a meaningful OS analysis was not feasible.

**Fig. 3:**
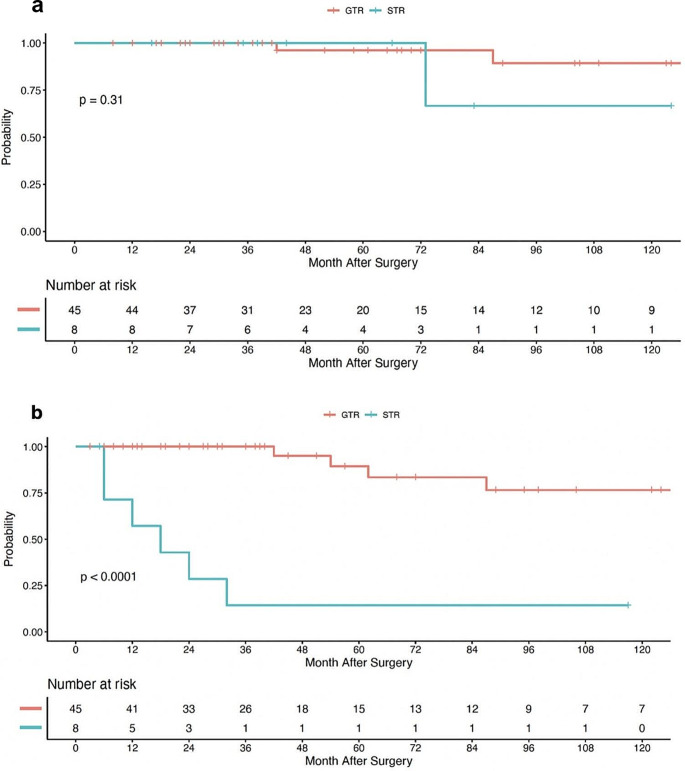
Kaplan-Meier curve for overall survival (**a**) and progression-free survival (**b**) for patients with non-myxopapillary pathology, stratified by extent of surgical resection

Considering a spinal ependymoma population with myxopapillary diagnoses removed, there was an increased disparity in OS when stratifying by EOR, however this finding was not statistically significant by Log-rank analysis in our sample size (*p* =.31). The 8-year OS rates for GTR and STR was 89% (95% CI, 76-100%) and 67% (95% CI, 36-100%), respectively, Fig. 3a. In terms of PFS, the GTR cohort benefited from superior outcomes compared to STR per Long-rank analysis (*p* <.001). Correspondingly, the 8-year PFS rates were 76% (95% CI, 59-100%) for the GTR cohort and 14% (95% CI, 2-88%) for the STR group, Fig. 3b.

## Discussion

This retrospective analysis including 69 patients diagnosed with a spinal ependymoma was the first large, contemporary, single institution study to our knowledge to investigate the relationship between the extent of primary surgical resection on both OS and PFS. Our results demonstrate that patients undergoing GTR experienced a significant increase in PFS compared to those with STR alone. Patients with a syrinx component to their pathology had improved GTR resection rates and a corresponding improvement in their PFS. Furthermore, patients without preoperative motor deficits and those who retained their ability to ambulate without assistance were more likely to have increased OS. Log-rank analysis did not reveal any significant relationship between extent of resection and OS.

The results derived from the current study are consistent with the existing body of literature relating to survival metrics for patients undergoing surgery for spinal ependymomas. In a retrospective multi-institutional analysis comprising of 126 spinal ependymoma patients surgically treated between 1953 and 2000, Abdel-Wahab et al. found OS rates via Kaplan-Meier estimates to be 91%, 84%, and 75% at 5-, 10- and 15-years, respectively. Disease progression occurred in 30.2% of patients at a median follow-up of 22 months with progression free survival rates at 5-, 10-, and 15-years estimated at 74%, 60%, and 35%, respectively [8]. More recently, Wostrack et al. reported 5-year PFS rates of 80% (median PFS not achieved) in an observational study of 158 patients undergoing primary ependymoma resection at a consortium of European centers from 2006 to 2013, although this study limited their focus to factors influencing radiographic progression only [19].

The current treatment paradigm for a spinal ependymoma diagnosis has been derived from a considerable collection of evidence investigating the factors associated with improvements in survival and progression free interval. Surgical resection for symptomatic patients has been a mainstay of therapy with a breadth of literature demonstrating improved outcomes compared to the natural history of the disease [20–22]. Adjuvant therapy with radiation and chemotherapy in select patients have also been studied with varying degrees of efficacy reported [11, 23–25]. Expectedly, we observed a greater percentage of patients from the STR receive adjuvant radiotherapy (STR: 64.3% vs. GTR: 7.3%; *p* <.001).

In patients undergoing primary surgical management, consistent evidence has demonstrated a positive association between GTR and PFS. An observational study of 118 patients with WHO grade 2 spinal ependymoma pathology undergoing resection in China from 2010 to 2016 found on univariate analysis that STR had 18.8 greater odds of recurrence compared to GTR [9]. Similarly, the aforementioned studies by Wostrack et al., Garces-Ambrossi et al., and Abdel-Wahab et al. all identified GTR as an independent predictor of improved PFS compared to STR, with Abdel-Wahab et al. additionally demonstrating that complete resection was significantly associated with improved OS [5, 8, 19, 26]. In the current study, we likewise found a strong association between extent of resection and disease recurrence, with 71.4% of STR patients experiencing progression compared to 9.1% in the GTR cohort (*p* <.01), a finding which was reinforced in the long-rank analysis (*p* <.001). It has been suggested that post-operative inflammation following GTR may eliminate or nullify microscopic remaining disease following ependymoma resection as has been demonstrated for other types of tumors, which in turn may augment the elimination of macroscopic disease in preventing recurrence [26, 27]. Alternatively, definitive conclusions pertaining to OS were difficult to detect in our cohort due to relatively low mortality event rates. To our knowledge, no recent study comprised of an exclusively modern patient cohort has shown a survival benefit of GTR over STR. This may in part be attributed to an improvement in the efficacy of adjuvant therapies and ancillary care. While a prior investigation utilizing a large cohort of patients followed by the SEER database from 2004 to 2014 demonstrated a survival benefit of GTR, this analysis included patients undergoing biopsy only (53.6%) and no surgery at all (7.2%), and did not have access to more granular clinical context [28].

Improved PFS following ependymoma resection has been associated with a number of other tumor specific characteristics including lower Ki-67% [9], lower tumor grade [8], Vimentin negative tumors [9], absence of MYCN amplification [29], and an identifiable tumor plane intraoperatively [26]. Interestingly, we found that patients with an associated syrinx were more likely to benefit from a GTR (*p* =.01), which could be attributed to more salient dissection planes intraoperatively. Moreover, patients with a grade 2 pathology were still more likely to have improved OS compared to grade 3 diagnoses (*p* <.05), despite accounting for the 2021 WHO update where myxopapillary ependymomas were upgraded to grade 2. Baseline functional status measured by various metrics such as low preoperative McCormick classification or Nurick grade have also been associated with improved postoperative outcomes [2, 4, 5, 30]. Similarly, we observed that patients who were able to ambulate without assistance benefitted from superior survival and disease progression metrics. This trend may in part be related to smaller and less symptomatic lesions having a higher likelihood of being amenable to GTR as both our current study and previous authors have demonstrated [19, 31], and emphasizes the importance of early diagnosis and management.

Disease recurrence rates in our myxopapillary cohort were similarly improved by a complete resection, a finding that is consistent with the existing literature [32]. We did however, observe a dip in the GTR rates when compared to the non-myxopapillary population, a phenomenon which may be attributed to the presence of a delicate tumor capsule which if violated, may result in dissemination of disease through the cerebrospinal fluid. Abdulaziz et al. demonstrated the importance of preserving the myxopapillary capsule in a series of surgical patients, where they observed increased disease recurrence rates in instances of capsule disruption, even if a complete radiographic resection was obtained [33]. Unfortunately, given the retrospective nature of our study, operative reports did not consistently comment on capsule integrity so this was not possible for us to study.

In the current study, we observed an overall GTR rate of 79.7%. While reported GTR rates for spinal ependymomas are varied, there has been an observed increase in the probability of GTR following the integration of what are now standard technologies for management of intramedullary lesions including the operating microscope (1957), MRI technology (1983), and the use of motor evoked potentials in spine surgery (1989) [16]. Specifically, a number of historical studies including patients prior to 1987 reported GTR rates between 50 and 60% [8, 10, 34], while more modern analyses have reported rates closer to 80% [19, 30]. Conducting quality survival analyses in a spinal ependymoma population comes with a number of challenges: it is a relatively rare and slow-growing pathology requiring large sample sizes with lengthy follow-up, which may result in a heterogenous cohort in the context of rapid advancements in operative technology and adjuvant therapies. Consequently, future study should be directed towards prospective multicenter efforts.

## Limitations

The findings and implications of this study should be evaluated within the context of its limitations. This survival analysis was conducted retrospectively and therefore is susceptible to the inherent limitations associated with a retrospective study design, including patients lost to follow-up and inconsistent or biased documentation. Additionally, as an observational study design, there was no standardized protocol for the timing or interpretation of postoperative imaging, which may have influenced the extent of resection designation or the identification of lesion recurrence. Finally, in order to make more decisive conclusions regarding independent predictors of OS and PFS, a multivariate regression analysis would have been preferred. However, as spinal ependymomas are a relatively rare disease and the objective was to study a contemporary cohort, the limited number of certain clinical events rendered this type of analysis impractical.

## Conclusions

In this large, contemporary single-center series of spinal ependymoma patients, we found patients undergoing GTR benefitted from superior PFS, but did not have an increase in OS. Preoperative ambulatory status was identified as a clinical correlate toward improvements in both survival and disease progression metrics. Finally, patients with an associated syrinx experienced higher GTR resection rates and likewise, improvement in their PFS. Future investigations should focus on prospective multicenter collaborations.

## Data Availability

The supporting data used to generate this manuscript may be available upon request.
